# *Populus trichocarpa* encodes small, effector-like secreted proteins that are highly induced during mutualistic symbiosis

**DOI:** 10.1038/s41598-017-00400-8

**Published:** 2017-03-23

**Authors:** Jonathan M. Plett, Hengfu Yin, Ritesh Mewalal, Rongbin Hu, Ting Li, Priya Ranjan, Sara Jawdy, Henrique C. De Paoli, George Butler, Tessa Maureen Burch-Smith, Hao-Bo Guo, Chun Ju Chen, Annegret Kohler, Ian C. Anderson, Jessy L. Labbé, Francis Martin, Gerald A. Tuskan, Xiaohan Yang

**Affiliations:** 1 INRA, UMR 1136 INRA-University of Lorraine, Interactions Arbres/Microorganismes, Laboratory of Excellence ARBRE, INRA-Nancy, 54280 Champenoux, France; 2 Hawkesbury Institute for the Environment, University of Western Sydney, Richmond, 2753 NSW Australia; 3 Biosciences Division, Oak Ridge National Laboratory, Oak Ridge, TN 37831 USA; 4 Department of Biochemistry & Cellular and Molecular Biology, University of Tennessee, Knoxville, TN 37996 USA

## Abstract

During symbiosis, organisms use a range of metabolic and protein-based signals to communicate. Of these protein signals, one class is defined as ‘effectors’, i.e., small secreted proteins (SSPs) that cause phenotypical and physiological changes in another organism. To date, protein-based effectors have been described in aphids, nematodes, fungi and bacteria. Using RNA sequencing of *Populus trichocarpa* roots in mutualistic symbiosis with the ectomycorrhizal fungus *Laccaria bicolor*, we sought to determine if host plants also contain genes encoding effector-like proteins. We identified 417 plant-encoded putative SSPs that were significantly regulated during this interaction, including 161 SSPs specific to *P. trichocarpa* and 15 SSPs exhibiting expansion in *Populus* and closely related lineages. We demonstrate that a subset of these SSPs can enter *L. bicolor* hyphae, localize to the nucleus and affect hyphal growth and morphology. We conclude that plants encode proteins that appear to function as effector proteins that may regulate symbiotic associations.

## Introduction

Symbiosis, defined as a durable interaction between two or more organisms, is a complex association that requires both physiological and morphological signaling, reception and alterations on the part of both organisms. Many fungal lineages within the pathogenic/mutualistic continuum have evolved elaborate protein-based signals to influence their hosts in order to support their metabolic requirements during symbiosis^1^. These proteins, called effectors, are typically fungal strain- or species-specific, usually ≤250 amino acids in size, and carry a secretion signal motif that may be cysteine-rich. Some examples of pathogenic effector proteins include: Avr3a of *Phytophthora infestans* ^2, 3^, the ChCEs of *Colletotrichum higginsianum* ^4^, RTP1 of *Uromyces fabae* ^5^ and the TIN2 of *Ustilago maydis* ^6^. More recently, effector proteins have also been characterized in mutualistic fungi. For example, MiSSP7 of the ectomycorrhizal (ECM) fungus *Laccaria bicolor* stabilizes the *Populus* jasmonic acid signaling repressor PtJAZ6^7^ while the SP7 protein of the arbuscular mycorrhizal fungus *Rhizophagus irregularis* (formerly *Glomus intraradices*) blocks the activity of ERF19^8^.

When viewed from the perspective of the fungal-encoded effector biology described to date, the outcome of symbiosis would appear to be heavily influenced by the colonizing fungus. Is this true, or could plants encode similar “effector-like” proteins that, in turn, exert a level of influence on the activity of the symbiotic associates? Previous research supporting the influence of plants over microbial biology suggests that: (1) plants utilize a number of secondary metabolites to affect rhizospheric and endopsheric microbes^9^, (2) general root exudates act as microbial chemoattractants^10^, (3) plants produce strigolactones which act as signals in initiating mycorrhizal formation^11^ and (4) flavonoids from plants function as antimicrobial compounds^12, 13^.

Recently, attention has turned to investigating proteins present in root exudates and their role in influencing plant-microbe interactions. Proteins, such as peptidases, hydrolases and defensins, have been implicated in affecting symbiosis^14–16^. There is also a precedent for plant small secreted proteins (SSPs; ≤250 amino acids) that are produced by roots and enter the cytosol of nitrogen-fixing bacteria during nodule formation to govern the outcome of these mutualistic interactions^17–20^. In total, 146 plant cysteine-rich SSPs have been identified^17, 19, 21^. It is generally understood that the discovery of SSPs in newly sequenced plant genomes has been hampered by the lack of gene annotation for proteins smaller than 100 amino acids (aa) due to a high false positive discovery rate from computational prediction of small open reading frames (ORFs) and a lack of homology across plant genomes which constrains *ab initio* gene calling^22, 23^.

The lack of SSP annotation is changing, however, due to the increasing availability of deep RNA-sequencing data. Recent re-annotation of the *Arabidopsis* and *Populus* genomes found evidence for 2,099 and 1,282 new small proteins, respectively^22, 24^. Moreover, mutation analysis of a number of these new *Arabidopsis* small proteins has demonstrated that a large portion play critical roles in plant developmental processes such as flowering, leaf development and overall plant structure^24^. However, the possibility that a number of these proteins could be secreted and function as effector proteins during symbiotic interactions is still uncertain. The aim of this study was to determine if: (1) *Populus* encoded effector-like proteins are regulated during mutualistic symbiosis with the ectomycorrhizal fungus *L. bicolor* and (2) whether some of these proteins might be able to enter the hyphae of *L. bicolor* and affect the growth of the fungus.

## Results

### Size bias in proteins predicted to be secreted during a mutualistic interaction

**Figure 1:**
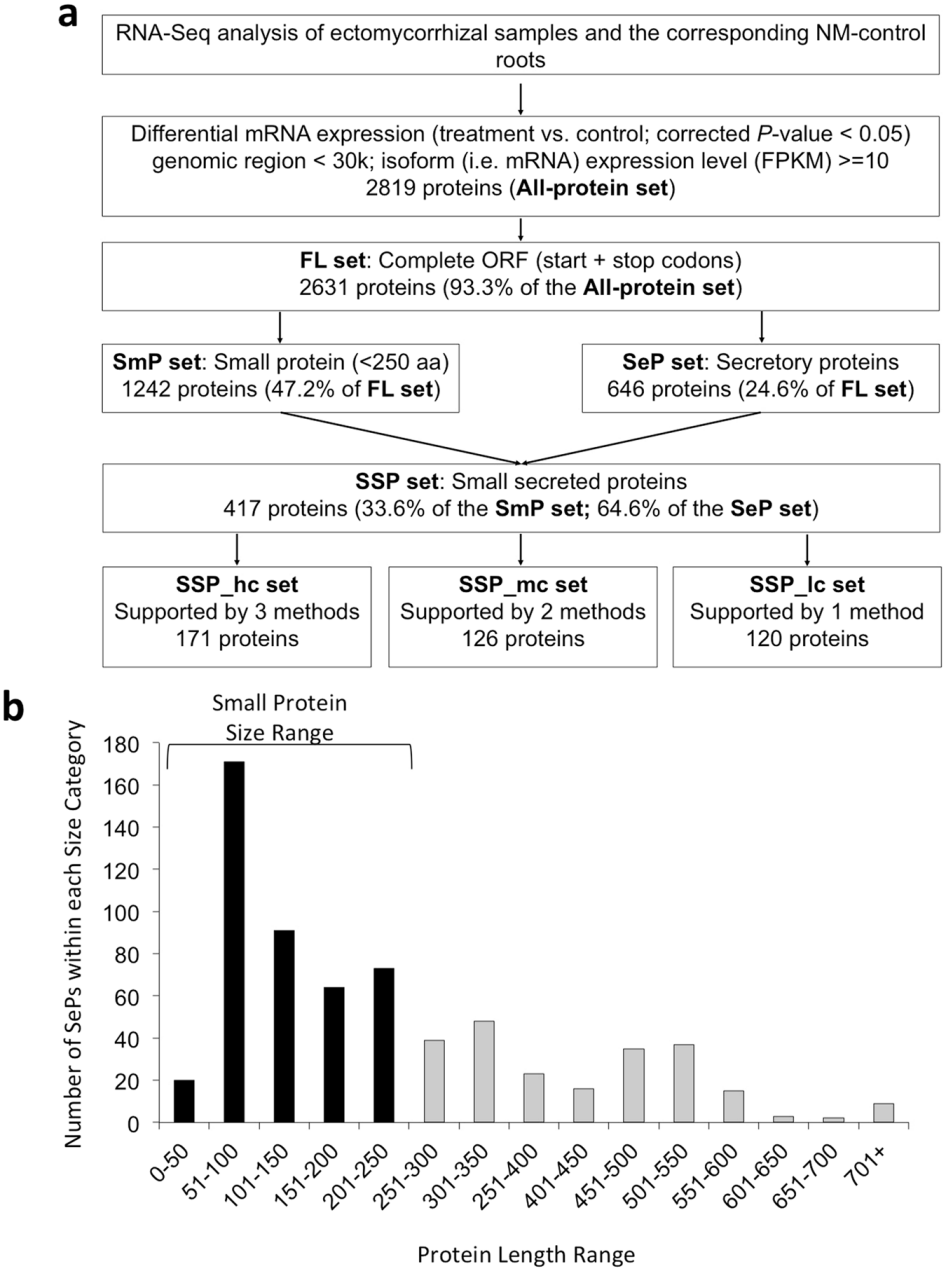
Characteristics of *Populus trichocarpa* genes differentially expressed during mutualistic symbiosis with *Laccaria bicolor*. (**a**) A computational pipeline for discovery of small protein-encoding genes in *P. trichocarpa* in response to *L. bicolor*. (**b**) Sequence length distribution of *P. trichocarpa* computationally-predicted secreted proteins expressed during mutualistic symbiosis with *L. bicolor*. ‘Small’ proteins are defined as ≤250 amino acids.

Using a computational pipeline for discovery of small protein-encoding genes based on transcriptomes analyzed by RNA-seq (Fig. 1a), we identified 2,819 *Populus* protein-encoding genes (designated as the ‘All-protein set’) that exhibited differential (p-value < 0.05) transcript abundance across all stages of mycorrhizal root tip development during symbiosis between *P. trichocarpa* and *L. bicolor*. The quantitative expression based on RNA-seq data was validated by quantitative RT-PCR analysis of 13 randomly-selected transcripts, which significantly correlated with the data obtained from the RNA-seq transcript analysis (p-value < 0.001; Supplementary Fig. S1; Supplementary Table S1). In the “All-protein set”, 2,631 transcripts (93.3%) contained a complete open reading frame (ORF) and were designated as the full-length (FL) set. In the FL set, 1,242 proteins (47.2%) satisfied the criteria for small proteins, i.e., ≤250 amino acids in length, and were designated as the “SmP set”. Given that SSPs are typically secreted, the predicted subcellular localizations of the FL set proteins were determined by three differential computational tools: LocTree2^25^, CELLO^26^ and YLoc^27^. From this analysis, 646 proteins in the FL set were predicted to code for secreted proteins (the SeP set). Within the SeP set, there was an over-representation of small proteins of ≤250 aa in length (64.6% of the SeP set), with the largest number of proteins falling within the size range of 51–100 aa (Fig. 1b). This subgroup of proteins was designated the SSP set (Supplementary Table S2).

This SSP set (417 proteins) was further divided into sub-sets based on three computational ‘confidence level’ approaches (see Methods section): the high confidence set (“SSP_hc set”; 171 proteins), the medium confidence set (“SSP_mc set”; 126 proteins) and the low confidence set (“SSP_lc set; 120 proteins; Fig. 1a; Supplementary Table S2). Proteins not found in the current *P. trichocarpa* genome annotation (37% of the SSP set) were named with the prefix ‘CUFF’ while proteins found in the current *Populus* genome annotation v3.0 (www.Phytozome.net) were designated with the prefix ‘Potri’. *In silico* analysis of the SSP set found that 79 of these SSPs contained predicted nuclear localization signals as predicted by using YLoc^27^ and CELLO^26^. Functional categorization of the differentially expressed SSPs with gene ontology (GO) biological process showed that these proteins are putatively involved in different biological pathways with varying biochemical activity (Supplementary Table S2). Overall, there was enrichment for stress-related GO terms, in particular the “response to fungus” category (Supplementary Table S3).

### Lineage- or genus-specific set of *P. trichocarpa* SSP genes

**Figure 2:**
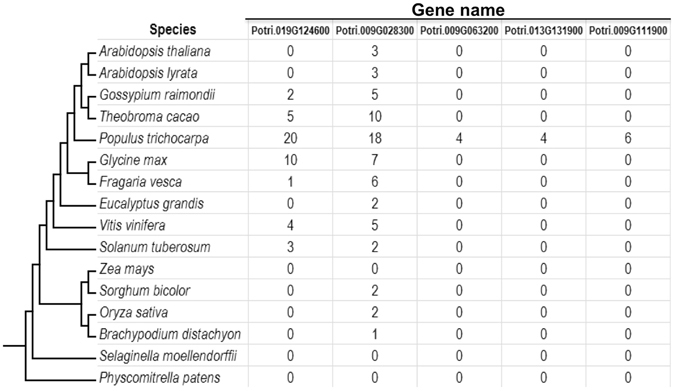
Lineage-specific expansion of small secreted proteins (SSPs) associated with *Populus trichocarpa*-*Laccaria bicolor* interaction. Due to space limitation, only five genes (Potri.019G124600, Potri.009G028300, Potri.009G063200, Potri.013G131900 and Potri.009G111900) showing lineage-specific expansion are shown in this figure. See Supplementary Tables S4 and S5 for details about the SSP genes showing lineage-specific expansion in *Populus* and closely related plant genera. The phylogenetic tree was adapted from http://phytozome.jgi.doe.gov.

Of the entire 417 *P. trichocarpa* SSP set, 39% (or 161 SSPs) appear to be specific to *Populus*, i.e., without homologs in the other 15 species examined (Fig. 2; Supplementary Table S4), while 15 SSPs have homologs found only within closely related plant genera (Supplementary Table S5). A comparison of SSPs between *P. trichocarpa* accessions (Nisqually-1 and 93–960; strong ECM host plants) and *P. deltoides* accessions (ILL-101 and D124; poor ECM host plants)^28^ showed that six SSPs predicted in the *P. trichocarpa* genomes were missing or truncated in the *P. deltoides* accessions (Table 1).

**Table 1:** SSP genes that have re-sequencing read coverage of >90% in *Populus trichocarpa* (“93–968” and “Nisqually-1”) and <50% in *P. deltoides* (“D124” and “ILL-101”).

Gene name	Confidence Group	Protein length, aa	Genomic location	*Populus trichocarpa*		*Populus deltoides*	
				Nisqually-1	93–968	D124	ILL-101
CUFF.22613.1	HC	216	Chr10:21706385–21717909	100	100	24	33
Potri.009G063200.1	HC	69	Chr09:6460994–6461507	100	99	14	33
CUFF.3798.1	LC	38	Chr01:47088970–47089866	99	100	0	0
Potri.004G136500.1	LC	249	Chr04:15714835–15715724	99	93	28	42
Potri.006G212200.1	LC	70	Chr06:22618830–22619398	100	98	13	45
CUFF.21535.1	MC	139	Chr01:48110837–48112147	99	98	15	23

Confidence levels HC, MC and LC represent three computationally-predicted SSP sets: the high confidence set (“SSP_hc set”), the medium confidence set (“SSP_mc set”) and the low confidence set (“SSP_lc set”), respectively.

### *Populus* SSPs have functional secretion signals

**Figure 3:**
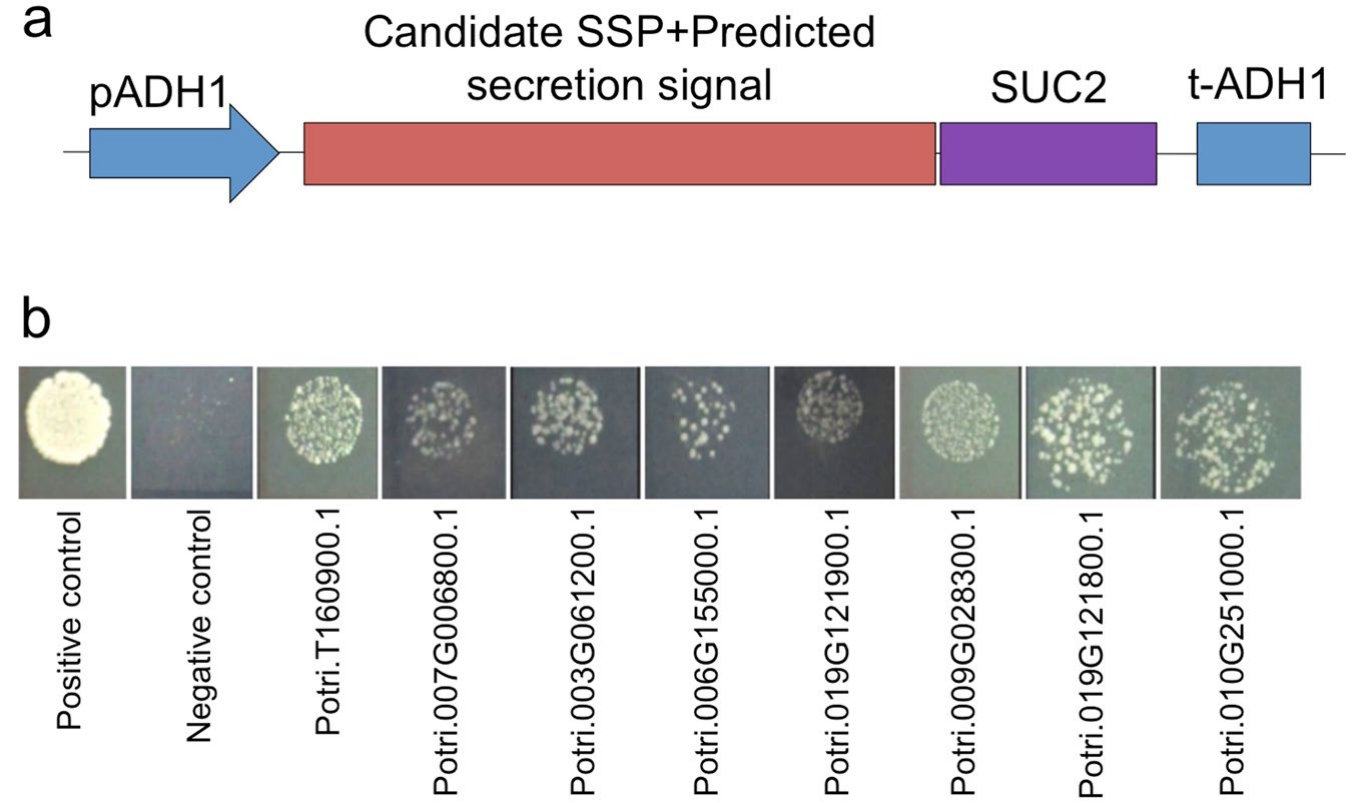
Testing of *Populus trichocarpa* small secreted proteins (SSPs) secretion using a yeast expression system. (**a**) Design of the yeast SUC2-SP vector used for the protein secretion assay^63^. The expression of the candidate *P. trichocarpa* SSP with a positive secretion signal will result in the secretion of the SUC2 protein allowing the growth of yeast on sucrose-containing media. (**b**) Representative examples of positive *P. trichocarpa* SSPs using the yeast secretion assay. The yeast strain growing on medium with glucose supplied was used as a positive control while the strain transformed with empty pGAD-SUC vector was used as a negative control.

To test the secretion of SSPs, a complementation assay in which the survival of the host depends on the secretion of the protein of interest was chosen. Forty full-length proteins (Supplementary Table S6), including their predicted secretion signals, were cloned upstream of a SUC2 invertase that lacked its native secretion signal. The recombinant genes were then expressed in the *Saccharomyces cerevisiae suc2* mutant that cannot grow on media containing sucrose as the sole energy source without the presence of a secreted SUC2 protein. We found that on average 15 of the 40 (38%) SSPs tested complemented the *suc2* mutation (Table 2; Fig. 3). Even though we used full-length genes, including the secretion signal in these tests, it is possible that the secondary structure of the small proteins masked the signal sequence and gave us a number of false negatives. Of the 40 SSPs tested, 16 were from the high confidence set (i.e., the SSP_hc set in Fig. 1a), and of those, 8 SSPs were able to able to rescue the mutant, yielding a 50% confirmation rate. In contrast, the medium confidence set of 14 SSPs (i.e., the SSP_mc set in Fig. 1a) and low confidence set of 10 SSPs (i.e., the SSP_lc set in Fig. 1a) each had a confirmation rate of around 30% (Table 2; Fig. 3). In summation, the signal peptides in some of the *Populus* SSPs were capable of directing secretion in a heterologous system.

**Table 2:** Percentage of *Populus* SSPs that encode secretion signals recognized in *S. cerevisiae.*

Confidence Level	Secretion Signals Tested	Number Positive for Secretion*	Percentage
HC	16	8	50.0
MC	14	4	28.6
LC	10	3	30.0
**Total/Average**	40	15	37.5

A subset from each confidence level of predicted *P. trichocarpa* SSPs was tested. Confidence levels HC, MC and LC represent three computationally-predicted SSP sets: the high confidence set (“SSP_hc set”), the medium confidence set (“SSP_mc set”) and the low confidence set (“SSP_lc set”), respectively.

*See Supplementary Table S6 for detailed results.

### *Populus* SSPs can enter *Laccaria bicolor* hyphae and accumulate in the nucleus

**Figure 4:**
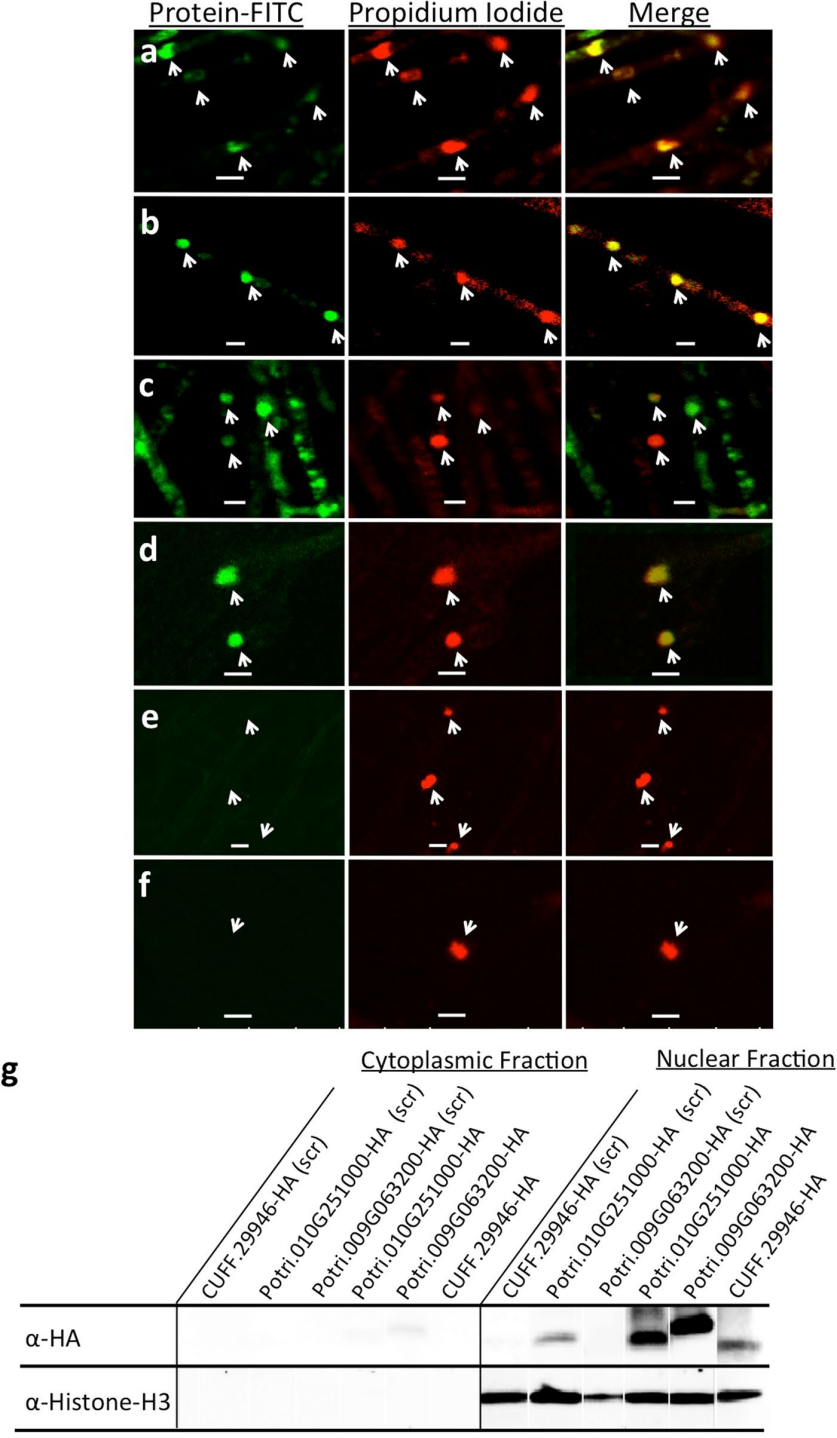
Uptake and sub-cellular localization of *Populus trichocarpa* small secreted proteins (SSPs) into *Laccaria bicolor* hyphae. *L. bicolor* colonies were incubated with 1 µM of either FITC-labelled SSP or FITC alone (control) in PBS (pH 7) for 4 hours after which they were rinsed briefly in PBS, fixed in 4% paraformaldehyde and then stained with 0.1% propidium iodide. Green signal indicates SSP-FITC (mature protein lacking secretion signal) florescent localization, red indicates propidium iodide fluorescence as a nucleus marker. Nuclei are marked by an arrow. (**a**) CUFF.29946; (**b**) Potri.009G063200; (**c**) Potri.010G251000; (**d**) Potri.007G006800; (**e**) Potri.019G121200; (**f**) FITC control; (**g**) Representative Western blot of cytoplasmic and nuclear protein extracted from *L. bicolor* exposed to either scrambled (scr) or native forms of CUFF.29946, Potri.009G063200 and Potri.010G251000 labeled with an HA tag. Probing for histone H3 shows purity of cytoplasmic fraction from nuclear protein.

In order to determine if *P. trichocarpa* SSPs could cross the *L. bicolor* hyphal membrane and accumulate within the fungal cells, we used five proteins (CUFF.29946, Potri.009G063200, Potri.010G251000, Potri.019G121200 and Potri.007G006800) produced by cell-free recombinant protein synthesis without the secretion signal and with an amino-terminal covalent addition of the FITC fluorescent molecule. Five biological replicates (i.e., independent *L. bicolor* colonies) were then treated with an individual protein or FITC alone on the leading edge of an *in vitro* fungal colony. Of the five FITC-tagged proteins tested, four (CUFF.29946, Potri.009G063200, Potri.010G251000 and Potri.007G006800) were able to enter the hyphae and concentrate in the nucleus (Fig. 4a–d). The fifth, Potri.019G121200, a negative control, did not localize to the nucleus of *L. bicolor* hyphae (Fig. 4e), indicating that the nuclear localization is not simply due to protein size. There was absence of fluorescent signal in the nuclei of hyphae treated with FITC alone (Fig. 4f). Localization of CUFF.29946, Potri.009G063200 and Potri.010G251000 was further verified using Western blotting of *L. bicolor* treated with *P. trichocarpa* SSPs modified with an HA tag rather than FITC (Fig. 4g). Further, we tested whether scrambling the amino acid sequence of these three proteins would affect their localization. We found that for Potri.009G063200 and CUFF.29946, scrambling the amino acid sequence led to reduced or absent import of the protein into *L. bicolor* hyphae. Scrambled Potri.010G251000 was still found to be imported into hyphae and localize to the nucleus, but at a reduced level (Fig. 4g). Therefore, some of the *P. trichocarpa* SSPs can enter *L. bicolor* hyphae.

### *Populus* SSPs can alter the development of multiple fungi

**Figure 5:**
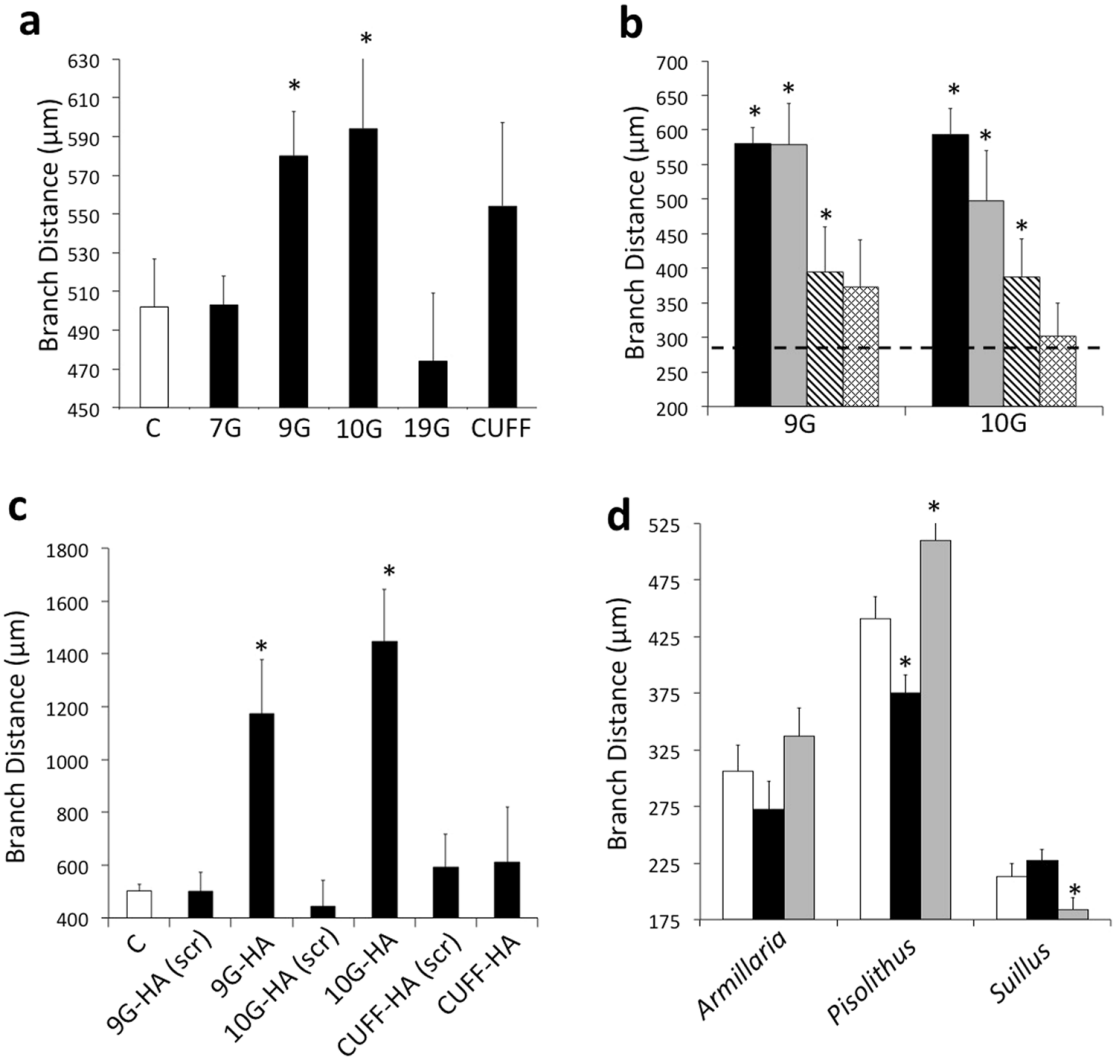
Effect of *Populus trichocarpa* small secreted proteins (SSPs) on hyphal branching. (**a**) Distance between hyphal branches in colonies of *L. bicolor* treated with 1 µM of FITC (C = control), Potri.007G006800 (7G), Potri.009G063200 (9G), Potri.010 G251000 (10G), Potri.019G121200 (19G) or with CUFF.29946.1 (CUFF). (**b**) Distance between hyphal branches in colonies of *L. bicolor* treated with 1 µM (black bars), 0.1 µM (grey bars), 0.01 µM (diagonal striped bars) or 0.001 µM (diamond pattern bars) Potri.009G063200 (9G) or Potri.010G251000 (10G) as compared to control treatment (dashed line, 1% DMSO). (**c**) Distance between hyphal branches in colonies of *L. bicolor* treated with 1 µM of either scrambled (scr) or native forms of Potri.009G063200 and Potri.010G251000 labeled with an HA tag as compared to control treatment (‘C’, 1% DMSO). (**d**) Impact of Potri.009G063200 (black bars) and Potri.010G25100 (grey bars) as opposed to control treatment (white bars) on the distance between hyphal branches of three different fungal species (*Armillaria luteobobulina*, *Pisolithus microcarpus*, *Suillus granulatus*) ± SE, *Significant difference from control (p < 0.05; Student’s t-test).

Finally, in pharmacological studies, where *L. bicolor* hyphae cultures were exposed to the four *Populus* SSPs that were able to move into the fungal nuclei, Potri.009G063200 and Potri.010G251000 significantly increased the distance between hyphal branches (p < 0.05; Fig. 5a). This effect was dose dependent (Fig. 5b). Scrambling of the amino acid sequence of both Potri.009G063200 and Potri.010G251000 blocked their ability to affect hyphal branching (Fig. 5c). We tested the ability of these two proteins to affect the growth of other ectomycorrhizal fungi; Potri.009G063200 significantly decreased branching distance in *Pisolithus microcarpus* (Boletales) while Potri.010G251000 increased branch distance of *P. microcarpus* and it significantly decreased branching distance in *Suillus granulatus* (Boletales) (p < 0.05; Fig. 5d).

**Figure 6:**
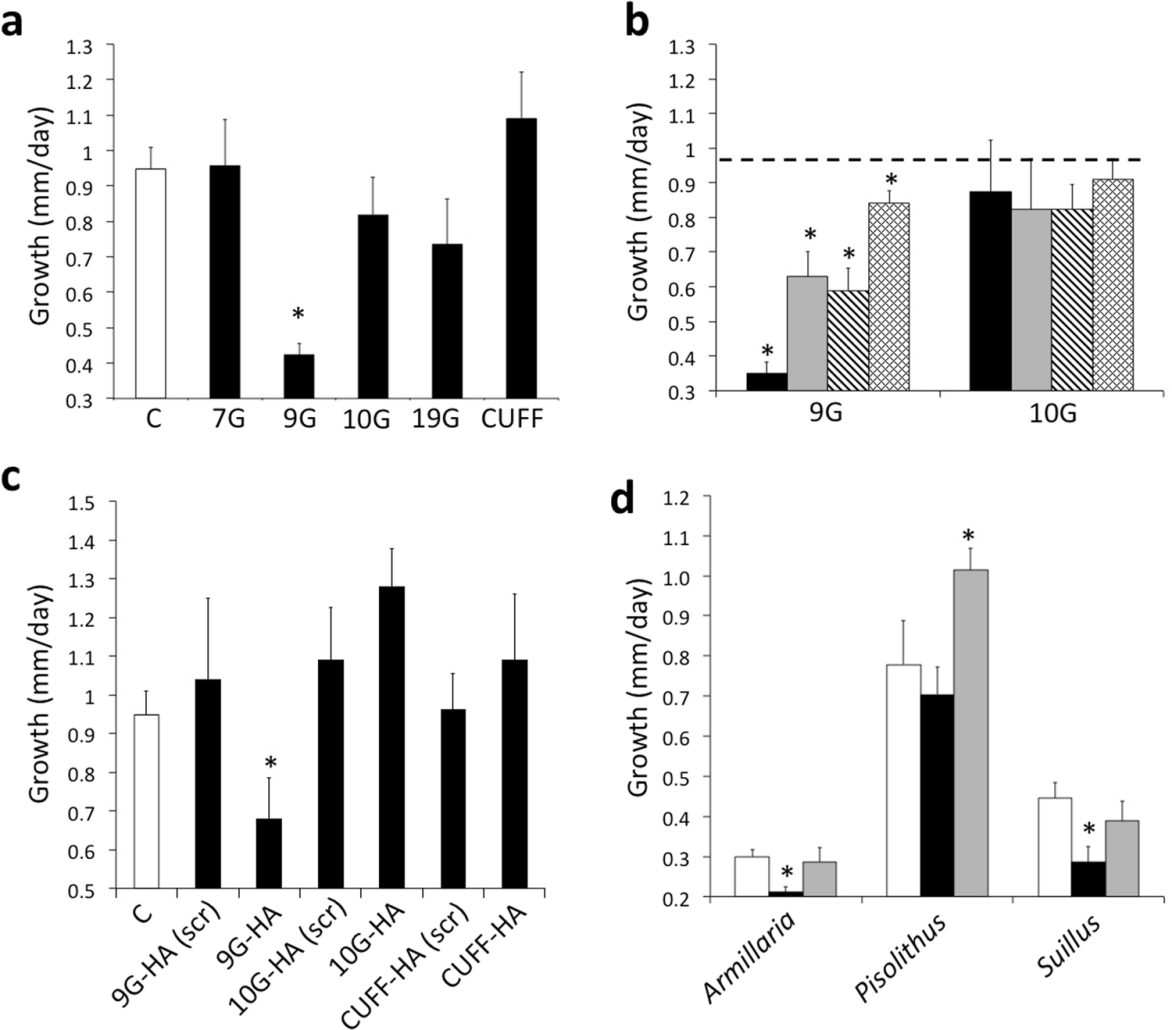
Effect of *Populus trichocarpa* small secreted proteins (SSPs) on hyphal growth rate. (**a**) Growth rate of *L. bicolor* hyphae treated with 1 µM of FITC (C = control), Potri.007G006800 (7G), Potri.009G063200 (9G), Potri.010G251000 (10G), Potri.019G121200 (19G) or with CUFF.29946.1 (CUFF). (**b**) Growth rate of *L. bicolor* hyphae treated with 1 µM (black bars), 0.1 µM (grey bars), 0.01 µM (diagonal striped bars) or 0.001 µM (diamond pattern bars) Potri.009G063200 (9G) or Potri.010G251000 (10G) as compared to control treatment (dashed line, 1% DMSO). (**c**) Growth rate of *L. bicolor* hyphae treated with 1 µM of either scrambled (scr) or native forms of Potri.009G063200 and Potri.010G251000 labeled with an HA tag as compared to control treatment (‘C’, 1% DMSO). (**d**) Impact of Potri.009G063200 (black bars) and Potri.010G25100 (grey bars) as opposed to control treatment (white bars) on the growth rate of hyphae of three different fungal species (*Armillaria luteobobulina*, *Pisolithus microcarpus*, *Suillus granulatus*) ± SE, *Significant difference from control (p < 0.05; Student’s t-test).

*L. bicolor* hyphae cultures were exposed to the four *Populus* SSPs that were able to move into the fungal nuclei also demonstrated that Potri.009G063200 significantly reduced the growth rate of *L. bicolor* hyphae (p < 0.05; Fig. 6a). This effect was dose dependent (Fig. 6b). Scrambling of the amino acid sequence of Potri.009G063200 blocked its effect on fungal growth rate (Fig. 6c). When both Potri.009G063200 and Potri.010G251000 were tested on other fungal species, Potri.009G063200 significantly reduced the growth of the pathogenic Agaricales *Armillaria luteobobulina* and *S. granulatus* (Fig. 6d). Potri.010G251000 increased the growth rate of the ectomycorrhizal *P. microcarpus*.

## Discussion

Mutualistic plant-microbe interactions are common among land plants. Interactions with mutualistic fungi and bacteria afford plant hosts enhanced stress tolerance^29–32^, increased growth rate and improved reproductive success^33, 34^. Our understanding of the mechanisms used by (1) plants to monitor, encourage and establish these relationships and/or (2) mutualistic organisms to colonize plants is limited. It has recently been established that mutualistic mycorrhizal fungi use SSPs to influence host functioning^7, 8, 35^. In the current study, we used computational predictions and experimental evidence to determine if the model perennial plant *P. trichocarpa* has an analogous genomic complement of effector-like SSPs. Genomic annotation of plant SSPs has lagged behind that of fungal SSPs. To address this limitation, we used a large RNA sequencing data-set sampled from different stages of *P. trichocarpa* root colonization by the mutualistic fungus *L. bicolor* to annotate novel *P. trichocarpa* SSPs. From a pool of differentially expressed transcripts, we identified 417 SSPs in *P. trichocarpa* expressed during mutualistic interaction with the ectomycorrhizal fungus *L. bicolor*. Of these 417 mycorrhiza-induced SSPs, 37% were undocumented in the current *P. trichocarpa* genome annotation v3.0 (www.Phytozome.net).

To functionally validate these proteins, we first showed that several of these small proteins contained verified secretion signals (Fig. 3), demonstrating the ability of these proteins to move across the membrane of the plant host cell. Secondly, we were able to demonstrate that four of six *P. trichocarpa* SSPs tested are capable of entering *L. bicolor* hyphae and localizing to the nuclei (Fig. 4). Interestingly, two of these *P. trichocarpa* SSPs significantly modified hyphal growth (Figs 5 and 6). A biological implication of this is that SSPs may be used to slow growth of fungal hyphae to prevent excessive growth within the root and thereby prevent parasitism. Alternatively, plant-based SSPs could be used to induce a switch in hyphal growth patterns from rhizospheric runner hyphae to aggregated hyphae on the root surface during the initial steps of symbiosis.

Plants may be exposed to hundreds of microbes at any given moment^36–38^. While only a small fraction of these organisms will attempt to colonize plant tissues, the plant must be able to distinguish between beneficial vs. detrimental microbes. The degree to which the plant is able to respond differently to organisms of different lifestyles (e.g., pathogenic vs. mutualistic) or between organisms of the same lifestyle (e.g., mutualistic vs. neutral) is poorly understood. Host species specificity in the transcriptomic response of the host to different microbes has been observed in the interaction between *Vitis vinifera* and *Burkholderia phytofirmans* or *Pseudomonas syringae* ^39^, between *Arabidopsis thaliana* and *Trichoderma asperelloides* or *P. syringae* ^40^, between *Hordeum vulgare* and *Piriformospora indica* or *Blumeria graminis* ^41^ and between *A. thaliana* and various viruses^42^, suggesting that the plant is able to tailor its SSP transcriptomic response to different lifestyles of microbes.

**Figure 7:**
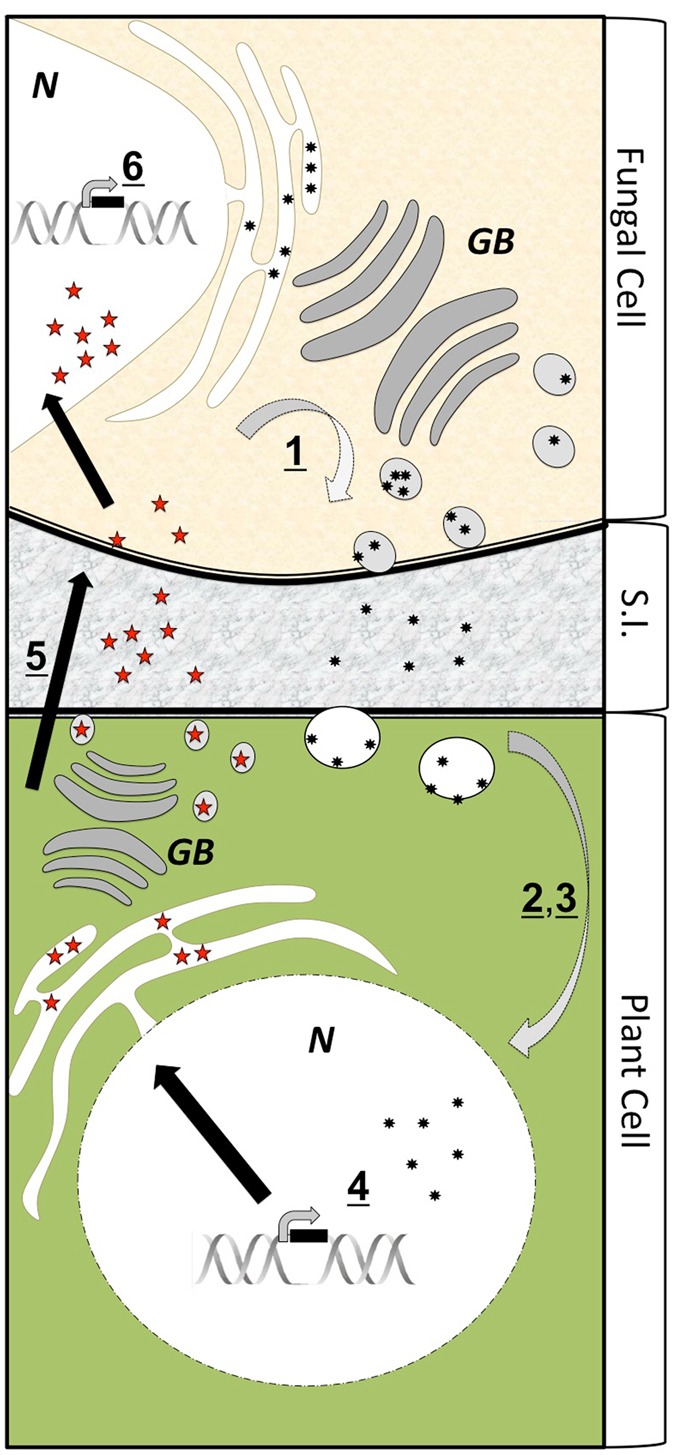
Proposed model of reciprocal small secreted proteins (SSP) feedback between ectomycorrhizal fungi and their host plants. During colonization of receptive plant hosts, fungal hyphae (brown cell) produce effector-like SSPs (black stars) that are secreted from the hyphal cell (#1). A portion of these proteins cross the symbiotic interface (grey shaded area) and enter the plant cell (green cell; #2) where they localize to different sub-cellular compartments (#3). These effectors may induce a change in the transcription of host genes (#4; adapted from Plett *et al.* ^35^). Plant cells, in turn, produce small effector-like SSPs (red stars) that are secreted, cross the symbiotic interface (#5) where some may enter the hyphal cell and localize to different sub-cellular compartments. Some effectors may induce a change in the transcription of host genes (#6). GB = Golgi bodies; N = nucleus; S.I. = symbiotic interface. Note: we do not currently know the priority of this signaling network (i.e. if the fungus signals and the plant responds or vice-versa). Rather, numbering here is used to enable description of the different steps in a feedback system that is likely reciprocal in nature.

Effector SSPs are a hallmark in the genomes of microbes that associate with plants^1^. These proteins are typically classified as ≤250 aa in length, are significantly regulated during symbiosis and coded by genes that show genomic evidence of rapid evolution^43, 44^. Upon secretion, these fungal effectors may remain external to plant cells or they may enter the plant cell. Regardless of their final localization, these proteins alter the functioning of the plant cell and their expression is generally considered crucial for the successful colonization of plant tissues^6, 35, 45, 46^. The discovery, described herein, of plant-encoded mycorrhiza-induced SSPs that are: (1) ≤250 aa in size, (2) lineage- or genera-specific, (3) highly expressed during symbiosis, (4) able to enter fungal hyphae and localize to the nucleus and (5) affecting fungal morphology strongly support the conclusion that plants encode proteins that are effector-like. This inference is supported by previous work in the legume:rhizobial interaction where small plant proteins have been reported to enter the symbiotic bacterium and affect its growth^17–21^. *Populus* can respond to mutualistic fungi by secreting their own effector-like proteins that might parallel the role of fungal effectors (Fig. 7). Our observation of effector SSPs in *Populus* implies a novel avenue, apart from the traditional immune response^44^, by which plants communicate with (or control) their mutualistic microbial partners.

Our data further supports previous hypotheses which has suggested that plants are not silent, or ‘naïve’, participants in mutualistic relationships by demonstrating that they too have the genetic tools in the form of effector-like proteins to communicate with and influence their microbial partners. These data are the first steps forward in understanding the role of plant SSPs as effectors in influencing symbiotic relationships. As more transcriptomic and genomic data related to SSPs becomes available, a more complete description of plasticity of plant responses to microbes will be possible. This research highlights the importance of the cross-talk between the plant and fungal effectors. Future studies on the fine-tuned balance between the number and level of the various released effectors could provide deeper insights into the development and the fitness of symbiosis.

## Methods

### Plant material, RNA extraction and sequencing

For colonization experiments, *L. bicolor* (Maire) P.D. Orton isolate S238N inoculum was grown on a substrate of peat moss:vermiculite (3:1) containing liquid Pachlewski medium for 3 months^47, 48^. Dormant hardwood stems from *Populus trichocarpa* clone 101–74 were collected from stool beds in January. Dormant stems were then cut into 25-cm cuttings with six to seven nodes per cutting. These cuttings were sealed in polyethylene bags and kept in cold storage (0 °C) until they were removed for rooting. The experiments in which these cuttings were used were then performed during the following year. Three node stem cuttings of *P. trichocarpa* clone 101–74 were pre-rooted for 1 week in a solution of 2.5 mM KNO_3_, 0.8 mM KH_2_PO_4_, 1 mM MgSO_4_ · 7 H_2_O, 2.3 mM Ca(NO_3_)_2_ · 4 H_2_O, 23 μM H_3_BO_3_, 4.6 μM MnCl_2_ · 4 H_2_O, 0.4 μM ZnSO_4_ · 7 H_2_O, 0.09 μM (NH_4_)_2_ MoO_4_, 0.18 μM CuSO_4_ · 5 H_2_O, 20 μM FeNa^.^EDTA, pH 5.8 and then planted in a 1 L-pot containing a 9:1 mixture (v:v) of Terra-Green to *L. bicolor* inoculum as previously described^49^. The colonization experiment was replicated twice (technical replicate) under climate controlled greenhouse conditions maintaining a 16-hr photoperiod at 22 °C.

Tissues used for transcriptomic profiling were sampled from fine roots of *P. trichocarpa* between 10 am and 2 pm each day to avoid confounding effects of circadian rhythm on gene expression. Plant host roots, grown in the same substrate and with the same nutrient regime but without fungal inoculum, were harvested as a non-mycorrhizal control for transcriptomic analyses. Three biological replicates of between 50–100 mg of colonized root tips (equivalent to approximately 100 colonized root tips) or of uncolonized lateral roots (control) were harvested at 2, 4 and 12 weeks after *L. bicolor* inoculation, washed thoroughly, snap frozen in liquid nitrogen and stored at −80 °C until total RNA extraction was performed using the RNAeasy kit (Qiagen) per the manufacturer’s instructions. An on-column DNA digestion step with DNAse I (Qiagen) was also included to avoid DNA contamination. RNA quality was verified using Experion HighSens capillary gels (Bio-Rad). For *P. trichocarpa* RNA sequencing (RNA-seq), three biological replicates of colonized or un-colonized tissues were pooled for sequencing. As mycorrhizal tissues are very recalcitrant to RNA extraction, quantities of RNA recovered were too low for quantities needed for RNA-seq analysis. Therefore, we amplified 250 ng total RNA using the Clonetech SMARTer amplification kit (Ozyme, St Quentin en Yvelines, France), according to manufacturer’s instructions and being sure to maintain the amplification in the logarithmic phase such that RNA pools were still proportional to original quantities of transcript as described previously^49^. Each pooled sample was sequenced (36-bp single-end reads) using Illumina GAII technology by IGA Technology services (Udine, Italy; GEO Accession Series GSE54789). Also, 100-bp single-end sequencing was also performed using Illumina HiSeq2000 platform (Beckman Coulter Genomics, Danvers, MA, USA) for samples collected 12 weeks after *Laccaria* inoculation (GEO Accession Series GSE75437).

### RNA-seq data analysis and prediction of *P. trichocarpa* small secreted proteins

The computational pipeline for RNA-seq data analysis and prediction of *P. trichocarpa* SSPs is illustrated in Fig. 1a. RNA-seq reads were mapped to the *P. trichocarpa* genome^50^ using TopHat^51^ and novel transcripts were discovered using Cufflinks^52, 53^. Differentially expressed genes between treatment (i.e., *P. trichocarpa* roots with *L. bicolor* ectomycorrhizas) and control (i.e., *P. trichocarpa* roots without *L. bicolor* inoculation) were identified using Cuffdiff^52^. The open reading frames (ORFs) were annotated using six-frame translation based on standard genetic code with a length range of 10–10,000 aa. The best ORF for each transcript was chosen based on those ORFs with the highest score in a BLASTp search using the default setting against the UniRef90 database (http://www.uniprot.org) or the longest ORF if there were no BLASTp hits.

The subcellular localizations of proteins were predicted using three different methods: (1) LocTree2^25^, (2) CELLO^26^ and (3) YLoc^27^ using default parameters. Proteins with full-length sequences of ≤250 aa were defined as small proteins (SmPs). The predicted small secreted proteins (SSPs) were divided into three sets: high-confidence set (SSP_hc) including proteins with subcellular localization as “secreted” predicted by method 1 and “extracellular space” by both method 2 and method 3; medium-confidence set (SSP_mc) proteins with subcellular localization as “secreted” predicted by method 1 and “extracellular space” by method 2 or method 3; and low-confidence set (SSP_lc) proteins with subcellular localization as “secreted” predicted by method 1, but no “extracellular” prediction by either method 2 or method 3.

### Protein domain and Gene Ontology analysis

A protein-domain search for all SSP sequences against the Pfam 28.0 database^54^ was performed with an E-value cutoff of 1e-10. Gene Ontology (GO) annotation was performed using Blast2GO with a BLASTp E-value hit filter of 1e-6, an annotation cutoff value of 55, and GO weight of 5^55^. GO enrichment analysis was performed using BiNGO^56^.

### Identification of homologs of *Populus* SSP genes in other plant species

The representative protein sequences (i.e., the longest protein sequence in case of multiple transcripts annotated for one gene locus) in 16 species (*Arabidopsis thaliana*, *Arabidopsis lyrata*, *Gossypium raimondii*, *Theobroma cacao*, *Populus trichocarpa*, *Glycine max*, *Fragaria vesca*, *Eucalyptus grandis*, *Vitis vinifera*, *Solanum tuberosum*, *Zea mays*, *Sorghum bicolor*, *Oryza sativa*, *Brachypodium distachyon*, *Selaginella moellendorffii*, *Physcomitrella patens*) were downloaded from Phytozome v9.0 (www.Phytozome.net). The homologs were identified using BLASTp^57^ with e-value cutoff of 1e-10.

### Comparative analysis of SSP genes among different *Populus* genotypes

*Populus* genome resequencing data^58, 59^ was used to reveal genomic differences in the region of selected genes. Two *P. trichocarpa* genotypes (Nisqually-1 and 93–968) and two *P. deltoides* genotypes (ILL-101 and D124) were selected for the analysis. The Illumina reads were aligned to the reference *P. trichocarpa* genome sequence^50^ to generate bam files using the MAQ software package^60^. Per base sequence coverage for mapped reads in the region corresponding to the selected genes were obtained using bedtools^61^.

### Testing of protein secretion using a yeast expression system

To construct a Gateway compatible vector for yeast secretion assay, a DNA fragment containing the *SUC* gene without signal peptide was synthesized by GeneScript (Piscataway, NJ, USA) and validated by sequencing. The DNA fragment was ligated into pGAD-424 vector by S*phI*. Candidate SSPs were cloned into the secretion vector, with the SSP gene fused with the N-terminus of *SUC* gene, by Gateway cloning and transformed into the *suc2* yeast mutant (strain ATCC-96100). Positive transformants were selected and validated on a synthetic dropout medium. Transformants were assayed on plates with glucose (10 mM) and sucrose (10 mM) as carbon supplies respectively and growth activity was measured after growing 2–3 days at 28 °C.

### *L. bicolo*r uptake of *P. trichocarpa* SSPs and growth analysis tests

In order to determine if *P. trichocarpa* SSPs could cross the *L. bicolor* hyphal membrane, five proteins (CUFF.29946, Potri.009G063200, Potri.010G251000, Potri.019G121200 and Potri.007G006800) were produced by cell-free recombinant peptide synthesis (Eurogentec) without the secretion signal and amino-terminal covalent addition of FITC using standard amino acid coupling techniques or with the addition of an HA tag (C-terminal addition of the following amino acid sequence: YPYDVPDYA). CUFF.29946, Potri.009G063200 and Potri.010G251000 were also produced in this method as a ‘scrambled’ (scr) version whereby the amino acids were randomly re-ordered and synthesized in a new sequence. Potri.019G121200 was used as a negative control, which contains a FASCICLIN-LIKE ARABINOGALACTAN PROTEIN 12 domain (www.phytozome.net), and it was predicted to be a cell adhesion protein and was not expected to be imported by fungal hyphae. The peptides were purified using HPLC fractionation to ensure the purity of the synthesis product. A control of the FITC fluorophore or of dilution buffer was also used throughout the experiments to ensure that this tag nor the buffer were altering our results.

To test subcellular localizations of these *P. trichocarpa* SSPs in *L. bicolor* hyphae and the role of these proteins in affecting fungal morphology (for *L. bicolor, P. microcarpus, A. luteobobulina, S. granulatus*), fungal colonies grown on cellophane membranes on ½ MMN agar plates at 25 °C until the colonies were 2–3 cm in diameter (2 weeks). Five biological replicates (i.e., independent colonies) were treated with an individual synthetic protein or FITC or buffer alone (controls) by dripping 10 µL of a solution of the protein (concentrations varying between 1 µM and 0.001 µM as outlined in the text) on the leading edge of the fungal colony. This treatment was repeated daily for four days after which the growth of the leading fungal edge and the hyphal branching were measured using ImageJ. The protein, FITC or buffer solutions were produced fresh daily and never re-used to avoid degradation of the protein.

For the localization experiment, the same aged *L. bicolor* colonies were treated by placing a 10 µL drop of either 1 µM protein or 1 µM FITC (PBS, pH7) on the leading edge of an in-tact, un-perturbed fungal colony grown as described above. After 4 hour of incubation, colonies were washed in excess PBS two times for five minutes each. The treated hyphae were then cut away from the rest of the colony and were fixed overnight in 4% (w/v) paraformaldehyde at 4 °C. Post fixing, samples were washed 3 × 10 min in PBS and then stained in 0.1% propidium iodide for 20 min then imaged using an inverted Leica TCS SP5 laser scanning confocal microscope. Fluorescent excitation was achieved using a 488 nm beam (10% power) for FITC and a 561 nm beam for propidium iodide. Emission was recovered between 510–530 nm for FITC and between 610–630 nm for propidium iodide. To corroborate fluorescent cell entry tests, *L. bicolor* colonies were exposed to HA-tagged proteins as above. Rather than fixing the samples, they were ground in liquid nitrogen and cytoplasmic and nuclear proteins were extracted as per Wang and colleagues^62^. Equal quantities of total protein were separated on a 1D Bio-Rad gradient gel (4–20%) and then transferred to a PVDF membrane. Membranes were blocked and then probed with either α-HA (to detect the presence of the SSP) or α-Histone H3 primary antibody (to ascertain the purity of the cytoplasmic vs. nuclear protein fraction). Bands were developed using the Clarity ECL Blotting substrate (Bio-Rad) as per manufacturer’s instructions and chemiluminescent images were taken using the Bio-Rad ChemiDoc Imaging system with an exposure of between 15–20 s.

## Electronic supplementary material

Supplementary_information

Supplementary Table S2

Supplementary Table S5
