# Supplementary material for: Populus trichocarpa encodes small, effector-like secreted proteins that are highly induced during mutualistic symbiosis
Source: Sci Rep. 2017 Mar 23;7:382. doi: 10.1038/s41598-017-00400-8 (PMC5428498; doi:10.1038/s41598-017-00400-8)
Supplement: Supplementary file 1 — Supplementary_information [file 41598_2017_400_MOESM1_ESM.doc]

**Supplementary Information**

***Populus* *trichocarpa* encodes small, effector-like secreted proteins that are highly induced during mutualistic symbiosis**

Jonathan M. Plett^1,2,*^, Hengfu Yin^3,*^, Ritesh Mewalal^3^, Rongbin Hu^3^, Ting Li^3^, Priya Ranjan^3^, Sara Jawdy^3^, Henrique C. De Paoli^3^, George Butler^4^, Tessa Maureen Burch-Smith^4^, Hao-Bo Guo^4^, Chun Ju Chen^3^, Annegret Kohler^1^, Ian C. Anderson^2^, Jessy L. Labbé^3^, Francis Martin^1^, Gerald A. Tuskan^3^, Xiaohan Yang^3^

^1^ INRA, UMR 1136 INRA-University of Lorraine, Interactions Arbres/Microorganismes, Laboratory of Excellence ARBRE, INRA-Nancy, 54280 Champenoux, France

^2^ Hawkesbury Institute for the Environment, University of Western Sydney, Richmond, 2753 NSW, Australia

^3^ Biosciences Division, Oak Ridge National Laboratory, Oak Ridge, TN 37831, USA

^4^Department of Biochemistry & Cellular and Molecular Biology, University of Tennessee, Knoxville, TN 37996, USA

**Supplementary Table S1.** Primer sequences used for quantitative RT-PCR analysis of gene expression.

| **Transcript** | **Forward primer** | **Reverse primer** |
| --- | --- | --- |
| CUFF.1886.1 | AGACCTTGGAGTGCACCTTG | CAAGAAATGTTGCAGGTTGG |
| CUFF.24568.1 | CCCAGACATCCCAAGAGAAA | CAGCTCCTGGAAAGGACAAG |
| CUFF.29946.1 | TTCCTGTGGAGACCAAATCC | CCAAGGCACAACAACAACAC |
| Potri.001G294800.1 | TCGGTGACATTTCAAGCATC | TGGAATCAAACCTTCCATCAC |
| Potri.003G072900.1 | TCCAAATCCCAGAAGGAGAA | TACATCGGAACCTCCACCTC |
| Potri.003G186400.2 | ATTCCCACGGGATGATTTG | GGAAACGGTGTCTCTGAAGG |
| Potri.004G095200.1 | CCCGAGTGAAGCTTTTGAGA | GAACGAATTAGAGGTAGGTTTCCA |
| Potri.006G021600.1 | GACGCTTGCTTTTGAGGAAC | AAGGAAGGGACAAAGCACAA |
| Potri.009G024300.1 | TCGCTCTGTTAGCATTCGAC | TGGAGACAACTCACCACCAG |
| Potri.010G109000.1 | ACTGCTGCTGTGAGTCTTGC | CTTGAGCTCGCGGTACTCTT |
| Potri.010G251000.1 | AATTCAGGCTGGAAGGGACT | ATCCTGCCCCTTGTTTCTTT |
| Potri.011G123600.1 | GGCATTAGCACCACCAAATC | CGGAAGTTAAACCACCGAAG |
| Potri.T131900.1 | TTACCTCCTGGTGGTGAGTTG | TCAGGCTTCTCAACGGTAGG |

**Supplementary Table S2.** *Populus trichocarpa* SSPs found to be differentially expressed in root tips undergoing colonization by *Laccaria bicolor* classified according to confidence level. Confidence levels HC, MC and LC represent three computationally-predicted SSP sets: the high confidence set (“SSP_hc set”), the medium confidence set (“SSP_mc set”) and the low confidence set (“SSP_lc set”), respectively.

(See separate EXCEL file)

**Supplementary Table S3.** Biological process enriched in *Populus* *trichocarpa* small protein-encoding genes up-regulated 12 weeks after inoculation with *Laccaria bicolor*.

| **GO-ID** | **Adjusted p-value** | **Number of genes** | **Description** |
| --- | --- | --- | --- |
| 50896 | 4.43E-14 | 268 | response to stimulus |
| 9719 | 4.43E-14 | 102 | response to endogenous stimulus |
| 9611 | 5.69E-13 | 43 | response to wounding |
| 6950 | 5.95E-13 | 195 | response to stress |
| 10200 | 3.55E-12 | 38 | response to chitin |
| 9725 | 2.15E-11 | 86 | response to hormone stimulus |
| 10033 | 2.57E-11 | 133 | response to organic substance |
| 42221 | 1.78E-09 | 175 | response to chemical stimulus |
| 9738 | 1.46E-07 | 24 | abscisic acid mediated signaling pathway |
| 9737 | 2.14E-07 | 47 | response to abscisic acid stimulus |
| 9755 | 3.24E-07 | 43 | hormone-mediated signaling pathway |
| 9753 | 4.27E-07 | 35 | response to jasmonic acid stimulus |
| 71215 | 5.27E-07 | 24 | cellular response to abscisic acid stimulus |
| 32870 | 5.91E-07 | 44 | cellular response to hormone stimulus |
| 71495 | 7.37E-07 | 50 | cellular response to endogenous stimulus |
| 9692 | 7.49E-07 | 15 | ethylene metabolic process |
| 9693 | 7.49E-07 | 15 | ethylene biosynthetic process |
| 43450 | 9.16E-07 | 15 | alkene biosynthetic process |
| 43449 | 9.16E-07 | 15 | cellular alkene metabolic process |
| 31407 | 2.01E-06 | 19 | oxylipin metabolic process |
| 9873 | 7.14E-06 | 14 | ethylene mediated signaling pathway |
| 9415 | 9.49E-06 | 35 | response to water |
| 42538 | 9.49E-06 | 17 | hyperosmotic salinity response |
| 9694 | 9.49E-06 | 17 | jasmonic acid metabolic process |
| 9414 | 1.34E-05 | 34 | response to water deprivation |
| 71369 | 2.60E-05 | 14 | cellular response to ethylene stimulus |
| 52548 | 3.72E-05 | 6 | regulation of endopeptidase activity |
| 10951 | 3.72E-05 | 6 | negative regulation of endopeptidase activity |
| 70887 | 4.45E-05 | 69 | cellular response to chemical stimulus |
| 2679 | 8.33E-05 | 11 | respiratory burst involved in defense response |
| 45730 | 8.46E-05 | 11 | respiratory burst |
| 9723 | 9.59E-05 | 24 | response to ethylene stimulus |
| 9743 | 9.59E-05 | 42 | response to carbohydrate stimulus |
| 9266 | 1.03E-04 | 57 | response to temperature stimulus |
| 52547 | 1.09E-04 | 6 | regulation of peptidase activity |
| 10466 | 1.09E-04 | 6 | negative regulation of peptidase activity |
| 160 | 1.19E-04 | 18 | two-component signal transduction system (phosphorelay) |
| 51346 | 1.51E-04 | 6 | negative regulation of hydrolase activity |
| 71310 | 3.01E-04 | 59 | cellular response to organic substance |
| 6970 | 3.41E-04 | 53 | response to osmotic stress |
| 9751 | 3.42E-04 | 26 | response to salicylic acid stimulus |
| 6972 | 3.85E-04 | 20 | hyperosmotic response |
| 9651 | 3.91E-04 | 50 | response to salt stress |
| 10286 | 6.04E-04 | 10 | heat acclimation |
| 50776 | 8.49E-04 | 24 | regulation of immune response |
| 2682 | 9.77E-04 | 24 | regulation of immune system process |
| 45088 | 1.55E-03 | 23 | regulation of innate immune response |
| 31408 | 1.55E-03 | 13 | oxylipin biosynthetic process |
| 42631 | 3.00E-03 | 10 | cellular response to water deprivation |
| 23033 | 3.09E-03 | 71 | signaling pathway |
| 45022 | 3.47E-03 | 3 | early endosome to late endosome transport |
| 6555 | 4.10E-03 | 17 | methionine metabolic process |
| 9620 | 5.76E-03 | 29 | response to fungus |
| 9628 | 6.34E-03 | 113 | response to abiotic stimulus |
| 19419 | 6.52E-03 | 3 | sulfate reduction |
| 9695 | 7.92E-03 | 11 | jasmonic acid biosynthetic process |
| 9408 | 8.32E-03 | 20 | response to heat |
| 31347 | 8.63E-03 | 27 | regulation of defense response |
| 9733 | 9.38E-03 | 24 | response to auxin stimulus |
| 15824 | 1.44E-02 | 7 | proline transport |
| 51336 | 1.51E-02 | 8 | regulation of hydrolase activity |
| 80134 | 1.59E-02 | 27 | regulation of response to stress |
| 9066 | 1.75E-02 | 17 | aspartate family amino acid metabolic process |
| 6952 | 1.75E-02 | 64 | defense response |
| 9867 | 1.75E-02 | 15 | jasmonic acid mediated signaling pathway |
| 9635 | 1.75E-02 | 4 | response to herbicide |
| 71395 | 1.77E-02 | 15 | cellular response to jasmonic acid stimulus |
| 9961 | 1.95E-02 | 3 | response to 1-aminocyclopropane-1-carboxylic acid |
| 9636 | 1.95E-02 | 6 | response to toxin |
| 9863 | 2.19E-02 | 16 | salicylic acid mediated signaling pathway |
| 9409 | 2.20E-02 | 35 | response to cold |
| 42398 | 2.26E-02 | 22 | cellular amino acid derivative biosynthetic process |
| 71446 | 2.31E-02 | 16 | cellular response to salicylic acid stimulus |
| 10363 | 2.54E-02 | 17 | regulation of plant-type hypersensitive response |
| 9862 | 2.85E-02 | 12 | systemic acquired resistance, salicylic acid mediated signaling pathway |
| 14070 | 3.06E-02 | 8 | response to organic cyclic substance |
| 9920 | 3.08E-02 | 3 | cell plate formation involved in plant-type cell wall biogenesis |
| 10941 | 3.09E-02 | 19 | regulation of cell death |
| 9607 | 3.21E-02 | 70 | response to biotic stimulus |
| 15804 | 3.25E-02 | 7 | neutral amino acid transport |
| 10083 | 3.25E-02 | 2 | regulation of vegetative meristem growth |
| 80135 | 3.67E-02 | 17 | regulation of cellular response to stress |
| 9061 | 3.76E-02 | 3 | anaerobic respiration |
| 42126 | 3.76E-02 | 3 | nitrate metabolic process |
| 42128 | 3.76E-02 | 3 | nitrate assimilation |
| 30968 | 3.97E-02 | 11 | endoplasmic reticulum unfolded protein response |
| 43455 | 4.00E-02 | 9 | regulation of secondary metabolic process |
| 6986 | 4.33E-02 | 11 | response to unfolded protein |
| 71445 | 4.33E-02 | 11 | cellular response to protein stimulus |
| 34620 | 4.33E-02 | 11 | cellular response to unfolded protein |
| 31326 | 4.70E-02 | 86 | regulation of cellular biosynthetic process |
| 6612 | 4.85E-02 | 17 | protein targeting to membrane |
| 9889 | 4.85E-02 | 86 | regulation of biosynthetic process |

**Supplementary Table S4.** *Populus trichocarpa*-specific SSPs that have no homologs in the other 15 plant species listed in Fig. 2. Confidence levels HC, MC and LC represent three computationally-predicted SSP sets: the high confidence set (“SSP_hc set”), the medium confidence set (“SSP_mc set”) and the low confidence set (“SSP_lc set”), respectively.

| **Gene ID** | **Amino Acid Length** | **Secretion Prediction Confidence Group** |
| --- | --- | --- |
| CUFF.10038.6 | 44 | LC |
| CUFF.10279.1 | 90 | LC |
| CUFF.10420.1 | 125 | MC |
| CUFF.10700.1 | 45 | MC |
| CUFF.11684.1 | 84 | MC |
| CUFF.11844.1 | 42 | LC |
| CUFF.12454.1 | 82 | MC |
| CUFF.12569.3 | 117 | HC |
| CUFF.12886.5 | 88 | HC |
| CUFF.13249.2 | 68 | MC |
| CUFF.14188.9 | 53 | MC |
| CUFF.14895.1 | 95 | MC |
| CUFF.15852.1 | 82 | HC |
| CUFF.16383.1 | 113 | LC |
| CUFF.16444.1 | 131 | HC |
| CUFF.1688.1 | 54 | HC |
| CUFF.16924.4 | 86 | HC |
| CUFF.17106.1 | 101 | MC |
| CUFF.17216.1 | 66 | MC |
| CUFF.17554.1 | 43 | HC |
| CUFF.17791.1 | 43 | LC |
| CUFF.17932.1 | 88 | MC |
| CUFF.1796.4 | 41 | MC |
| CUFF.18022.1 | 65 | MC |
| CUFF.18106.3 | 47 | HC |
| CUFF.18107.1 | 53 | MC |
| CUFF.18776.1 | 43 | LC |
| CUFF.20038.1 | 27 | MC |
| CUFF.21515.2 | 51 | HC |
| CUFF.21535.1 | 139 | MC |
| CUFF.21634.1 | 45 | LC |
| CUFF.21759.1 | 122 | LC |
| CUFF.22071.2 | 114 | MC |
| CUFF.22807.1 | 41 | MC |
| CUFF.22989.1 | 58 | MC |
| CUFF.24109.1 | 34 | LC |
| CUFF.24567.1 | 70 | HC |
| CUFF.24568.1 | 69 | HC |
| CUFF.2473.1 | 71 | HC |
| CUFF.24893.1 | 65 | LC |
| CUFF.25880.1 | 66 | MC |
| CUFF.2617.4 | 111 | MC |
| CUFF.26634.1 | 54 | HC |
| CUFF.26723.1 | 119 | LC |
| CUFF.26977.13 | 44 | MC |
| CUFF.27026.1 | 82 | MC |
| CUFF.27547.2 | 80 | HC |
| CUFF.27647.1 | 171 | HC |
|  |  |  |
| CUFF.28656.1 | 70 | HC |
| CUFF.29498.1 | 115 | MC |
| CUFF.29744.1 | 122 | HC |
| CUFF.29851.1 | 115 | MC |
| CUFF.29852.1 | 90 | MC |
| CUFF.30052.1 | 138 | HC |
| CUFF.3036.1 | 32 | MC |
| CUFF.30385.2 | 46 | HC |
| CUFF.30913.1 | 55 | HC |
| CUFF.31175.1 | 246 | LC |
| CUFF.3184.1 | 78 | HC |
| CUFF.32130.1 | 79 | HC |
| CUFF.32309.1 | 41 | LC |
| CUFF.32382.1 | 74 | MC |
| CUFF.32738.1 | 180 | LC |
| CUFF.33144.2 | 111 | MC |
| CUFF.33352.1 | 41 | HC |
| CUFF.33709.1 | 61 | LC |
| CUFF.3426.1 | 70 | LC |
| CUFF.34488.1 | 78 | MC |
| CUFF.34488.2 | 78 | MC |
| CUFF.34682.1 | 40 | LC |
| CUFF.34985.1 | 64 | LC |
| CUFF.35079.1 | 201 | LC |
| CUFF.35225.2 | 53 | MC |
| CUFF.35245.1 | 90 | HC |
| CUFF.35476.1 | 60 | LC |
| CUFF.3605.6 | 69 | LC |
| CUFF.3798.1 | 38 | LC |
| CUFF.3826.1 | 83 | MC |
| CUFF.4249.1 | 68 | MC |
| CUFF.4250.1 | 53 | MC |
| CUFF.4569.1 | 65 | LC |
| CUFF.4579.1 | 48 | MC |
| CUFF.4580.2 | 63 | HC |
| CUFF.4636.1 | 57 | LC |
| CUFF.4703.2 | 87 | HC |
| CUFF.4996.1 | 70 | HC |
| CUFF.53.1 | 58 | MC |
| CUFF.5421.1 | 91 | MC |
| CUFF.5646.1 | 58 | HC |
| CUFF.639.1 | 59 | MC |
| CUFF.7133.1 | 55 | MC |
| CUFF.7355.1 | 61 | HC |
| CUFF.8220.1 | 80 | HC |
| CUFF.8732.3 | 103 | LC |
| CUFF.9161.1 | 206 | LC |
| Potri.001G042100.1 | 154 | HC |
| Potri.001G125100.1 | 114 | MC |
| Potri.001G172000.1 | 94 | MC |
| Potri.001G174000.1 | 104 | MC |
| Potri.001G198800.1 | 102 | HC |
| Potri.001G239700.1 | 70 | HC |
| Potri.001G400200.1 | 66 | LC |
| Potri.002G068100.1 | 67 | HC |
| Potri.002G088800.1 | 81 | MC |
| Potri.002G112400.1 | 100 | HC |
| Potri.002G131800.1 | 72 | MC |
| Potri.002G223100.2 | 164 | LC |
|  |  |  |
| Potri.002G226300.1 | 74 | HC |
| Potri.002G257600.1 | 107 | MC |
| Potri.003G084300.1 | 117 | MC |
| Potri.003G154000.1 | 106 | HC |
| Potri.003G214400.1 | 117 | HC |
| Potri.004G068800.1 | 63 | HC |
| Potri.004G069000.1 | 67 | HC |
| Potri.004G113000.1 | 121 | MC |
| Potri.004G169000.1 | 108 | HC |
| Potri.004G169000.2 | 86 | MC |
| Potri.004G202300.1 | 81 | HC |
| Potri.004G202600.1 | 81 | LC |
| Potri.004G236100.1 | 193 | LC |
| Potri.005G122000.1 | 69 | LC |
| Potri.005G123600.1 | 73 | HC |
| Potri.006G038300.1 | 89 | MC |
| Potri.007G103500.1 | 79 | MC |
| Potri.008G045500.1 | 194 | LC |
| Potri.008G082000.1 | 58 | LC |
| Potri.008G087700.3 | 70 | LC |
| Potri.008G179400.1 | 75 | MC |
| Potri.008G179400.2 | 75 | MC |
| Potri.008G179400.3 | 75 | MC |
| Potri.009G063000.1 | 70 | HC |
| Potri.009G063200.1 | 69 | HC |
| Potri.009G111800.1 | 76 | HC |
| Potri.009G111900.1 | 90 | HC |
| Potri.009G168700.1 | 186 | MC |
| Potri.010G118500.1 | 116 | MC |
| Potri.010G220800.1 | 126 | LC |
| Potri.011G096800.1 | 73 | HC |
| Potri.011G108100.1 | 109 | MC |
| Potri.012G007100.1 | 79 | LC |
| Potri.012G014800.1 | 71 | MC |
| Potri.013G031900.1 | 74 | HC |
| Potri.013G033500.1 | 70 | MC |
| Potri.013G131900.1 | 70 | LC |
| Potri.014G156600.1 | 74 | HC |
| Potri.016G013000.1 | 144 | LC |
| Potri.016G019100.1 | 70 | LC |
| Potri.018G010500.1 | 53 | HC |
| Potri.018G072100.1 | 93 | LC |
| Potri.018G076800.1 | 93 | LC |
| Potri.018G078800.1 | 78 | HC |
| Potri.019G016000.1 | 116 | MC |
| Potri.019G016100.1 | 106 | HC |
| Potri.019G017300.1 | 110 | MC |
| Potri.019G121800.1 | 69 | MC |
| Potri.T027800.1 | 88 | LC |
| Potri.T051400.1 | 141 | MC |
| Potri.T051500.1 | 141 | MC |
| Potri.T089100.1 | 76 | MC |
| Potri.T127600.1 | 78 | MC |
| Potri.T160900.1 | 91 | HC |

**Supplementary Table S5.** *Populus trichocarpa* SSPs that have homologous genes in relatively closely related genus listed in Fig. 2.

(See separate EXCEL file)

**Supplementary Table S6.** List of *Populus trichocarpa* SSPs used for secretion test in yeast. Confidence levels HC, MC and LC represent three computationally-predicted SSP sets: the high confidence set (“SSP_hc set”), the medium confidence set (“SSP_mc set”) and the low confidence set (“SSP_lc set”), respectively.

| **Gene ID** | **Confidence Level (HC, MC, LC)** | **Secretion confirmed in yeast** |
| --- | --- | --- |
| Potri.T160900.1 | HC | Positive |
| Potri.005G167900.1 | HC | Negative |
| Potri.003G171800.1 | HC | Negative |
| Potri.007G006800.1 | HC | Positive |
| Potri.001G119000.1 | HC | Negative |
| Potri.004G169000.1 | HC | Positive |
| Potri.015G128700.1 | HC | Negative |
| Potri.005G123600.1 | HC | Negative |
| Potri.003G061200.1 | HC | Positive |
| Potri.003G134600.1 | HC | Negative |
| Potri.T155100.1 | HC | Negative |
| Potri.006G155000.1 | HC | Positive |
| Potri.009G063200.1 | HC | Negative |
| CUFF.29946.1 | HC | Positive |
| Potri.019G121900.1 | HC | Positive |
| Potri.002G161900 | HC | Positive |
| Potri.016G131800.1 | MC | Negative |
| Potri.009G028300.1 | MC | Positive |
| Potri.019G017300.1 | MC | Negative |
| Potri.012G023400.1 | MC | Negative |
| Potri.019G121800.1 | MC | Positive |
| Potri.014G087600.1 | MC | Negative |
| Potri.010G251000.1 | MC | Positive |
| Potri.014G095000.1 | MC | Negative |
| Potri.016G131400.2 | MC | Negative |
| Potri.018G007300.1 | MC | Negative |
| Potri.016G131700.1 | MC | Negative |
| Potri.008G179400.3 | MC | Positive |
| Potri.005G127900.3 | MC | Negative |
| Potri.014G121900.6 | MC | Negative |
| Potri.004G236100.2 | LC | Negative |
| Potri.016G131600.1 | LC | Negative |
| Potri.001G449900.1 | LC | Negative |
| Potri.002G155300.1 | LC | Negative |
| Potri.014G140200.1 | LC | Negative |
| Potri.T043800.1 | LC | Negative |
| CUFF.16383.1 | LC | Positive |
| Potri.008G045500.1 | LC | Negative |
| CUFF.2412.1 | LC | Positive |
| Potri.003G186400.3 | LC | Positive |

**Supplementary Figure S1.** Validation of RNAseq based quantitative expression data with quantitative RT-PCR.
